# Hetrombopag treatment for immune thrombocytopenia in pregnancy refractory to corticosteroids and intravenous immunoglobulin: a case report

**DOI:** 10.3389/fmed.2025.1528131

**Published:** 2025-02-18

**Authors:** Luyi Pang, Feng Yu, Xiaoyang Yang

**Affiliations:** Department of Hematology, Haikou Municipal Hospital and Central South University Xiangya School of Medicine Affiliated Haikou Hospital, Haikou, China

**Keywords:** pregnancy, immune thrombocytopenia, hetrombopag, recombinant human thrombopoietin, case report

## Abstract

Pregnancy can lead to the recurrence or exacerbation of immune thrombocytopenia (ITP). Currently, first-line treatments of low-dose corticosteroids or intravenous immunoglobulin are considered safe and effective for both pregnant women and fetuses. However, there is no well-established treatment option for patients who are refractory to these medications. Herein, we report a case of a 31-year-old pregnant woman with recurrent and refractory ITP who was safely and effectively treated with hetrombopag, with no influence on the infant's platelet count. This case explores a new treatment option for the management of recurrent and refractory ITP in pregnancy.

## Introduction

Platelet reduction occurs in ~1 out of 10 pregnancies, with immune thrombocytopenia (ITP) accounting for 1%−4% of cases of thrombocytopenia in pregnancy, which is ~10 times the incidence rate in the general population (1–3). However, the limited investigation of effective and safe medications for pregnant women with ITP and their fetuses poses a significant challenge. Currently, glucocorticoids are the preferred initial therapy for these patients. Due to their side effects, glucocorticoids should be used at the lowest effective dose to maintain platelet counts in a safe range. The recommended starting dosage of prednisone is 10 mg/day, which can be modified as appropriate (1–3). Fetal side effects of glucocorticoids are rare, as they are largely metabolized in the placenta. Additionally, intravenous immunoglobulin (IVIG) is recommended for pregnant patients who either do not respond to or cannot tolerate glucocorticoid treatment, or when a rapid increase in platelet count is necessary. IVIG can be administrated at a dosage of 1 g/kg per day for 2 days or 400 mg/kg per day for 5 days, either alone or in combination with low-dose prednisone. If initial therapy with glucocorticoids and IVIG fails, no suitable second-line treatment options are available (4). Recombinant human thrombopoietin (rhTPO) is a treatment option for pregnant patients during the third trimester in certain regions, such as China (5, 6). rhTPO, with its large molecular weight, could not cross the placenta and thus has no impact on the fetus (3). However, it can lead to the production of TPO antibodies, limiting its use in early pregnancy or long-term application. Rituximab, splenectomy, and thrombopoietin receptor agonists (TPO-RAs) may be considered treatment options during pregnancy for recalcitrant disease when first-line treatments have failed. However, these interventions may have unknown maternal and fetal adverse effects (1). Some studies have suggested that thrombopoietin receptor agonists (TPO-RAs), such as eltrombopag or romiplostim, may be safe and effective for the treatment of recurrent and refractory ITP in pregnant women who were unresponsive to IVIG or low-dose corticosteroids (7, 8).

The Chinese-developed small molecule non-peptide TPO-RA, hetrombopag, has been modified from eltrombopag to enhance efficacy while reducing hepatotoxicity (9, 10). Hetrombopag was approved by the National Medical Products Administration (NMPA) in June 2021 for the treatment of adult patients with chronic primary ITP who have had suboptimal responses to corticosteroids or immunoglobulins, but there have been no reports on its use for treating ITP in pregnancy to date.

We report for the first time a case of pregnant woman with ITP who was refractory to IVIG, low-dose corticosteroids and rhTPO, and who was treated with hetrombopag.

## Case report

In July 2023, a 29-year-old female patient came to our hospital with a low platelet count (5 × 10^9^/L) at 5 weeks of gestation. The patient had been diagnosed with ITP in 2021 and had received dexamethasone (orally, 40 mg per day). However, the disease recurred with 2 weeks. High-dose dexamethasone treatment was administered again. Three months after the initial treatment, ITP recurred and the patient received rhTPO combined with high-dose dexamethasone. The physician suggested second-line treatments such as TPO-RA or CD20 monoclonal antibodies or splenectomy, but the patient declined due to financial reasons. Alternatively, prednisone (1 mg/kg/day) was given for 2 weeks, followed by a gradual taper.

After being diagnosed with ITP in July, 2023, the patient received prednisone (15 mg/kg/day) and IVIG (400 mg/kg/day) in a 4 week cycle. At 8 weeks of gestation, the patient's platelet count remained below 10 × 10^9^/L. Therefore, rhTPO was added to the treatment regimen. Subsequently, the platelet count increased to 13 × 10^9^/L.

**Figure 1:**
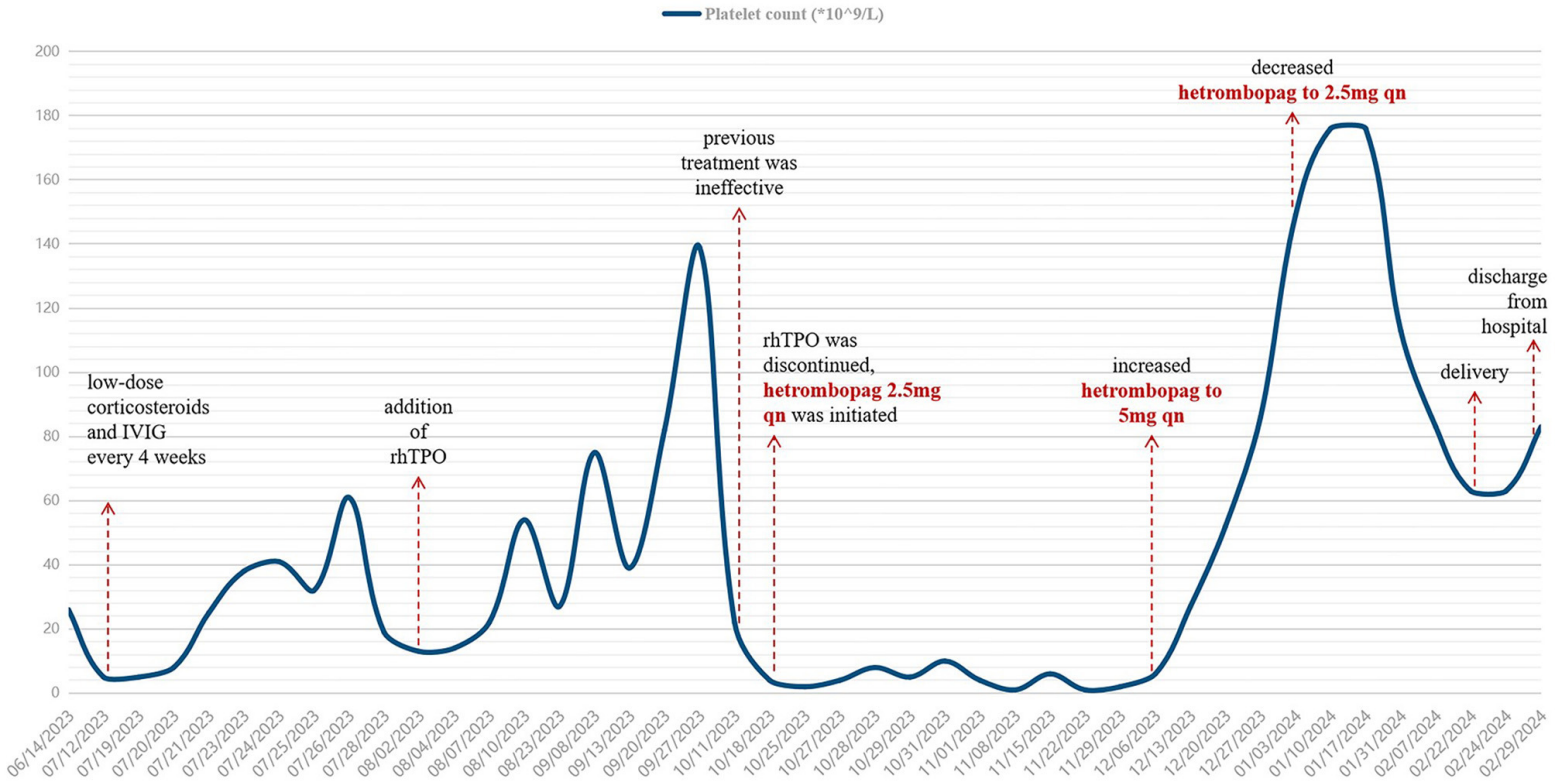
Changes in platelet counts of the patient from the time of treatment during pregnancy. IVIG, intravenous immunoglobulin; rhTPO, recombinant human thrombopoietin. After multiple lines of treatment, this patient with ITP in pregnancy had been refractory to corticosteroids or IVIG. After the dose of hetrombopag was increased to 5 mg qn, the patient's platelet count increased significantly, and then was maintained at a safe level.

In October 2023, when the patient was at 18 weeks of gestation, the platelet count fell to 4 × 10^9^/L, which was suspected to be due to the production of rhTPO autoantibodies, therefore, rhTPO was discontinued. With the patient's informed consent, oral hetrombopag treatment was initiated. The platelet count did not increase with an initial dose of 2.5 mg qn. The dose was then increased to 5 mg qn, and by December 2023, the platelet count increased to 27 × 10^9^/L. In January 2024, the patient's platelet count reached 149 × 10^9^/L, and the dose of hetrombopag was reduced to 2.5 mg qn. The changes in platelet counts of the patient were seen in Figure 1.

On February 22, 2024, the patient was admitted to the obstetrics department with Candida albicans infection, premature rupture of membranes, threatened preterm labor, and pregnancy complicated by ITP. On February 22, oxytocin was used for induction of labor, and a preterm female infant was delivered smoothly with Apgar scores of 10 at 1, 5, and 10 min. The infant's weight was 2,230 g and her length was 44 cm. The infant had a normal platelet count at birth (146 × 10^9^/L) and on day 5 (139 × 10^9^/L) and was subsequently discharged in good health.

## Discussion

In this case, the patient was treated with hetrombopag for 4 months and low-dose corticosteroids for over 6 months. Due to personal hygiene reasons and long-term corticosteroid therapy, the patient developed Candida albicans vaginitis, leading to preterm birth. However, the fetus only had elevated bilirubin and was discharged in good health after receiving symptomatic therapy. After the dose of hetrombopag was increased to 5 mg qn, the patient's platelet counts increased and maintained at a safe level. Overall, the treatment with hetrombopag was safe and effective. Therefore, for pregnant women with ITP, it is recommended that the initial dose of hetrombopag be 2.5 mg per day, and whether a higher dose can achieve better efficacy and safety requires further confirmation.

Hetrombopag is a novel small molecule non-peptide TPO-RA that can bind to the transmembrane region of the TPO receptor (TPO-R), activating the TPO-R dependent STAT, PI3K, and ERK signal transduction pathways, stimulating the proliferation and differentiation of megakaryocytes, and promoting platelet production. Based on the structure of eltrombopag, hetrombopag has been optimized with a heterocyclic carboxylic acid replacing the biphenyl structure to reduce liver toxicity, and a benzene and saturated carbon ring replacing xylene, enhancing lipophilicity and improving efficacy. The starting dose of herombopag is generally 2.5 mg per day, and the dose can be adjusted based on platelet counts, with a maximum dose of 15 mg per day. According to the drug's prescribing information, a dose of 200 mg/kg/day decreased the number of corpora lutea and implantation sites in female mice, lowered the number of live fetuses, and increased post-implantation loss rates. The no observed adverse effect level was 50 mg/kg/day. The dose of herombopag used in this patient was far below the levels shown to affect fetal development, and the patient did not breastfeed. During treatment, the drug's side effects were monitored, and the dosage of herombopag was adjusted based on platelet counts to achieve the goal of ensuring safe delivery while minimizing the dosage. Platelet counts were maintained at 50–80 × 10^9^/L. The patient did not experience any side effects mentioned in the drug instructions (11).

From the perspective of protein level pharmacological research, hetrombopag has a significantly stronger phosphorylation effect on various signaling proteins than eltrombopag at the same dose. Compared with rhTPO, it shows a more sustained and stable effect on downstream signal phosphorylation levels (12). Several phase III clinical studies have shown its good therapeutic effect in ITP (9, 13). Moreover, in phase II clinical studies of SAA refractory to immunosuppressive therapy (14), the results showed good safety of hetrombopag. The drug was approved for marketing by the NMPA's priority review procedure on June 17, 2021, and was approved for the treatment of adult patients with chronic ITP who have had suboptimal responses to corticosteroids and immunoglobulins, as well as adult patients with SAA refractory to immunosuppressive therapy.

Pregnant women with ITP, especially those in the late stage of pregnancy, may become insensitive corticosteroids and IVIG. Moreover, most drugs used to treat ITP may complicate pregnancy-related issues, such as gestational diabetes, hypertension, and psychiatric disorders, potentially impacting fetal development and growth (15, 16). Currently, no standard second-line treatment has been established for pregnant women with ITP, resulting in a therapeutic dilemma (17). For pregnant women, high-dose glucocorticoids can harm the fetus. Anti-(Rh)D can cause severe hemolytic reactions in both the fetus and the mother and should only be used in patients who are refractory to glucocorticoids and IVIG. However, there is no Anti-(Rh)D in China yet. Experience with cyclosporine and azathioprine in pregnancy is largely derived from case series involving patients with rheumatologic disorders and solid-organ transplantation (18). Theoretically, they will pass through the placenta and may affect the baby, so it is not recommended. Splenectomy can be considered for pregnant ITP patients who are unresponsive or intolerant to available drugs, but splenectomy later during pregnancy becomes a greater risk as the uterus becomes larger. Splenectomy is ideally performed in the first half of pregnancy when other options have not worked. However, most patients refuse splenectomy. CD20 monoclonal antibody can cross the placenta, and transfer from mother to fetus increases with gestational age. A review of a global rituximab drug safety database identified 153 pregnant patients associated with rituximab exposure. Of these, 90 (59%) resulted in live births; 22 (14%) were associated with premature birth; 11 neonates (7%) had hematologic abnormalities; one neonate died at age 6 weeks; and two infants had congenital malformations (19). Most of these pregnant patients were affected by teratogenic agents and maternal conditions (e.g., lymphoma). Vinca alkaloids, cyclophosphamide, and danazol are not recommended during pregnancy (19).

Data on the safety of TPO-RAs such as romiplostim and eltrombopag during pregnancy are limited, but emerging information suggests they do not cause a high rate of adverse events (7, 20). Severe neonatal thrombocytopenia (platelet counts < 20 × 10^9^/L) occurs in 1%−4% of ITP pregnancies, while moderate neonatal thrombocytopenia (platelet counts < 50 × 10^9^/L) occurs in 9% of ITP pregnancies. The application of TPO-RA may help alleviate thrombocytopenia in infants. Common side effects of TPO receptor agonists include mild headache, arthralgia, nasopharyngitis, fatigue, diarrhea, nausea, and increased marrow reticulin. These side effects are generally mild and rarely cause treatment discontinuation. Abnormalities of liver function tests (elevated aspartate aminotransferase, alanine aminotransferase, and bilirubin levels) have been observed in Asians receiving eltrombopag therapy but not with romiplostim or hetrombopag (7). Autoantibodies against romiplostim or thrombopoietin may develop but rarely reported in hetrombopag or eltrombopag. Although the 2019 American Society of Hematology (ASH) guidelines do not recommend TPO-RA, their use is individualized based on availability, cost, patient comorbidities, and preferences (21). Whether they have an impact on the fetus needs further research. TPO receptor expression has been demonstrated on the leukemic cells of patients with acute myelogenous cell, but not in lymphoid malignancies, myeloproliferative neoplasms, or other non-hematologic malignancies. Therefore, the application of hetrombopag does not inhibit platelet killing in pregnant women, but only increases platelet production. After the pregnant woman gave birth normally, the patient's platelets still did not recover. Although the application of hetrombopag can penetrate the placenta and may cause an increase in infant platelets, this phenomenon has not been observed in the present case. Nevertheless, this hypothesis needs to be verified in future studies.

Although TPO-RA drugs have not been approved by any regulatory agency for use in pregnancy, there are some retrospective studies and case reports focus on the use of TPO-RA in pregnant women with ITP (20, 22). A review summarized the safety and efficacy of TPO-RA. The data were collected from 24 studies involving pregnant women with ITP (22 cases using romiplostim, 21 cases using eltrombopag, and 2 cases using both). Before TPO-RA treatment, these pregnant women had experienced a median of three prior lines of therapy. Eventually, a platelet response (>30 × 10^9^/L) was observed in 86.7% of the cases (including 66.7% complete response >100 × 10^9^/L), and the efficacy of eltrombopag and romiplostim was similar (87.0% and 83.3%, *p* = 0.99). Maternal safety was preserved, and no thrombotic events occurred. One-third of newborns had thrombocytopenia, including one case of grade 3 intracranial hemorrhage, and three newborns had thrombocytosis. No other adverse events attributable to TPO-RA were observed (22). Another study reviewed 10 case reports and a cohort study with the use of TPO-RA in pregnant women with ITP, showing that TPO-RA drugs were relatively safe at any time in pregnancy, with no reports of congenital malformations. However, there seems to be an association between the use of eltrombopag in the first and second trimesters and low birth weight (20).

There was also a multicenter observational retrospective study reporting 15 pregnant women with ITP (17 pregnancies; 18 newborns), of which 8 cases were in the eltrombopag group and 7 cases in the romiplostim group. Two cases had secondary ITP. The median duration of TPO-RA exposure in pregnancy was 4.4 weeks (1–39 weeks). In 17 pregnancies, 10 cases (58%) started TPO-RA treatment as an indication for preparation for delivery, 4 cases were chronic refractory symptomatic ITP, and 3 cases were receiving eltrombopag treatment at the beginning of pregnancy. In terms of safety, except for one case of neonatal thrombocytosis, no maternal thrombotic events occurred, and no fetal or neonatal complications related to TPO-RA were observed. A total of 77% of the cases responded to TPO-RA, most of them responded to TPO-RA in combination with another ITP treatment (70% of responders) (23).

## Conclusion

In summary, for pregnant women with recurrent and refractory ITP who are unresponsive to corticosteroids and IVIG, the off-label use of TPO-RA treatment before delivery seems to be safe and effective for both mothers and the newborns. We report for the first time a case of hetrombopag treatment for pregnant women with recurrent and refractory ITP, providing a new treatment option for such patients. More prospective studies are needed in the future to explore the safety and efficacy of TPO-RA for treating ITP in pregnancy.

## Data Availability

The raw data supporting the conclusions of this article will be made available by the authors, without undue reservation.

## References

[1] Zhang H Shi L Shang H Yang H. Immune thrombocytopenic purpura and maternal and neonatal outcomes during pregnancy: a systematic review and meta-analysis. Am J Reprod Immunol. (2024) 92:e70008. 10.1111/aji.70008 39498982

[2] Bussel JB Knightly KA. Immune thrombocytopenia (ITP) in pregnancy. Br J Haematol. (2024) 204:1176–7. 10.1111/bjh.19230 38263610

[3] Ruszala M Poniedziałek-Czajkowska E Mierzynski R Wankowicz A Zamojska A Grzechnik M. Thrombocytopenia in pregnant women. Ginekol Pol. (2021) 92:587–90. 10.5603/GP.a2021.0147 34541631

[4] Dahiphale SM Dewani D Agrawal M Dahiphale JM Jyotsna G Saloni. Navigating primary immune thrombocytopenia during pregnancy with management strategies and considerations: a comprehensive review. Cureus. (2024) 16:e67449. 10.7759/cureus.67449 39314573 PMC11417416

[5] Lin J Wang T-F Huang M-J Huang H-B Chen P-F Zhou Y. Recombinant human thrombopoietin therapy for primary immune thrombocytopenia in pregnancy: a retrospective comparative cohort study. BMC Pregn Childb. (2023) 23:820. 10.1186/s12884-023-06134-y 38012579 PMC10680270

[6] Yu J Miao P Qian S. Application of recombinant human thrombopoietin in pregnant women with immune thrombocytopenia: a single-center experience of four patients and literature review. J Int Med Res. (2023) 51:3000605231187950. 10.1177/03000605231187950 37548331 PMC10408329

[7] Bussel JB Cooper N Lawrence T Michel M Vander Haar E Wang K. Romiplostim use in pregnant women with immune thrombocytopenia. Am J Hematol. (2023) 98:31–40. 10.1002/ajh.26743 36156812 PMC10091785

[8] Shibata S Misugi T Nakane T Hino M Tachibana D. Use of eltrombopag for the first trimester pregnancy complicated with refractory idiopathic thrombocytopenic purpura: a case report and literature review. Cureus. (2022) 14:e22505. 10.7759/cureus.22505 35371812 PMC8958988

[9] Mei H Liu X Li Y Zhou H Feng Y Gao G. Switching from eltrombopag to hetrombopag in patients with primary immune thrombocytopenia: a *post-hoc* analysis of a multicenter, randomized phase III trial. Ann Hematol. (2024) 103:2273–81. 10.1007/s00277-024-05826-5 38842566 PMC11224074

[10] Shen N Qiao J Jiang Y Yan J Wu R Yin H. Thrombopoietin receptor agonists use and risk of thrombotic events in patients with immune thrombocytopenic purpura: a systematic review and meta-analysis of randomized controlled trials. Biomed Rep. (2024) 20:44. 10.3892/br.2024.1732 38357229 PMC10865300

[11] Syed YY. Hetrombopag: first approval. Drugs. (2021) 81:1581–5. 10.1007/s40265-021-01575-1 34357499

[12] Xie C Zhao H Bao X Fu H Lou L. Pharmacological characterization of hetrombopag, a novel orally active human thrombopoietin receptor agonist. J Cell Mol Med. (2018) 22:5367–77. 10.1111/jcmm.13809 30156363 PMC6201220

[13] Mei H Liu X Li Y Zhou H Feng Y Gao G. A multicenter, randomized phase III trial of hetrombopag: a novel thrombopoietin receptor agonist for the treatment of immune thrombocytopenia. J Hematol Oncol. (2021) 14:37. 10.1186/s13045-021-01047-9 33632264 PMC7905908

[14] Peng G He G Chang H Gao S Liu X Chen T. A multicenter phase II study on the efficacy and safety of hetrombopag in patients with severe aplastic anemia refractory to immunosuppressive therapy. Ther Adv Hematol. (2022) 13:20406207221085197. 10.1177/20406207221085197 35371427 PMC8972928

[15] Fogerty AE Kuter DJ. How I treat thrombocytopenia in pregnancy. Blood. (2024) 143:747–56. 10.1182/blood.2023020726 37992219

[16] Pishko AM Marshall AL. Thrombocytopenia in pregnancy. Hematol Am Soc Hematol Educ Prog. (2022) 2022:303–11. 10.1182/hematology.2022000375 36485110 PMC9820693

[17] Ümit EG Demir AM Ar MC Ayer M Ayli M Karakuş V. Management of primary immune thrombocytopenia: Turkish modified Delphi-based consensus statement for special considerations. Turk J Haematol. (2024) 41:141–5. 10.4274/tjh.galenos.2024.2024.0101 38801066 PMC11589376

[18] Kaushansky K Lichtman MA Prchal JT Levi MM Burns LJ. Williams Hematology, 10th ed, Chapter 116 Thrombocytopenia. New York, NY: McGraw-Hill Education (2021). p. 2093–124.

[19] Chakravarty EF Murray ER Kelman A Farmer P. Pregnancy outcomes after maternal exposure to rituximab. Blood. (2011) 117:1499–506. 10.1182/blood-2010-07-295444 21098742

[20] Howaidi J AlRajhi AM Howaidi A AlNajjar FH Tailor IK. Use of thrombopoietin receptor agonists in pregnancy: a review of the literature. Hematol Oncol Stem Cell Ther. (2022) 15:1–6. 10.1016/j.hemonc.2021.05.004 34153229

[21] Sahi PK Chandra J. Immune thrombocytopenia: American Society of Hematology guidelines, 2019. Indian Pediatr. (2020) 57:854–6. 10.1007/s13312-020-1966-8 32999115

[22] Rottenstreich A Bussel JB. Treatment of immune thrombocytopenia during pregnancy with thrombopoietin receptor agonists. Br J Haematol. (2023) 203:872–85. 10.1111/bjh.19161 37830251

[23] Michel M Ruggeri M Gonzalez-Lopez TJ Alkindi S Cheze S Ghanima W. Use of thrombopoietin receptor agonists for immune thrombocytopenia in pregnancy: results from a multicenter study. Blood. (2020) 136:3056–61. 10.1182/blood.2020007594 32814348

